# Enterococcus faecalis Responds to Individual Exogenous Fatty Acids Independently of Their Degree of Saturation or Chain Length

**DOI:** 10.1128/AEM.01633-17

**Published:** 2017-12-15

**Authors:** Holly E. Saito, John R. Harp, Elizabeth M. Fozo

**Affiliations:** a Department of Microbiology, University of Tennessee, Knoxville, Tennessee, USA; North Carolina State University

**Keywords:** Enterococcus faecalis, fatty acids, daptomycin, cerulenin, membrane stress

## Abstract

Enterococcus faecalis is a commensal of the human gastrointestinal tract that can persist in the external environment and is a leading cause of hospital-acquired infections. Given its diverse habitats, the organism has developed numerous strategies to survive a multitude of environmental conditions. Previous studies have demonstrated that E. faecalis will incorporate fatty acids from bile and serum into its membrane, resulting in an induced tolerance to membrane-damaging agents. To discern whether all fatty acids induce membrane stress protection, we examined how E. faecalis responded to individually supplied fatty acids. E. faecalis readily incorporated fatty acids 14 to 18 carbons in length into its membrane but poorly incorporated fatty acids shorter or longer than this length. Supplementation with saturated fatty acids tended to increase generation time and lead to altered cellular morphology in most cases. Further, exogenously supplied saturated fatty acids did not induce tolerance to the membrane-damaging antibiotic daptomycin. Supplementation with unsaturated fatty acids produced variable growth effects, with some impacting generation time and morphology. Exogenously supplied unsaturated fatty acids that are normally produced by E. faecalis and those that are found in bile or serum could restore growth in the presence of a fatty acid biosynthetic inhibitor. However, only the eukaryote-derived fatty acids oleic acid and linoleic acid provided protection from daptomycin. Thus, exogenous fatty acids do not lead to a common physiological effect on E. faecalis. The organism responds uniquely to each, and only host-derived fatty acids induce membrane protection.

**IMPORTANCE** Enterococcus faecalis is a commonly acquired hospital infectious agent with resistance to many antibiotics, including those that target its cellular membrane. We previously demonstrated that E. faecalis will incorporate fatty acids found in human fluids, like serum, into its cellular membrane, thereby altering its membrane composition. In turn, the organism is better able to survive membrane-damaging agents, including the antibiotic daptomycin. We examined fatty acids commonly found in serum and those normally produced by E. faecalis to determine which fatty acids can induce protection from membrane damage. Supplementation with individual fatty acids produced a myriad of different effects on cellular growth, morphology, and stress response. However, only host-derived unsaturated fatty acids provided stress protection. Future studies are aimed at understanding how these specific fatty acids induce protection from membrane damage.

## INTRODUCTION

Although Enterococcus faecalis is a natural commensal of the mammalian intestine, it can persist in a variety of external environments and is a common hospital-acquired infectious agent associated with a variety of pathologies that include surgical wound infections, bacterial endocarditis, and bacteremia. Contributing to its ability to thrive as a commensal, an environmental organism, and a pathogen, E. faecalis is resilient in the presence of a variety of stressors, including pH and osmotic alterations, temperature shifts, nutrient limitation, and many others (1). Further, the organism is inherently tolerant and/or resistant to a variety of antibiotics, making successful treatment of infections difficult (2).

A critical component for the survival of many bacterial species during environmental stress is alteration of their membrane fatty acid composition, and this is particularly common in response to temperature changes (reviewed in references 3 and 4). For example, many bacteria, including the model organisms Escherichia coli and Bacillus subtilis, will increase the proportion of unsaturated membrane fatty acids in response to decreasing temperatures, presumably to maintain membrane fluidity (reviewed in reference 3). We have shown that like many well-characterized microorganisms, E. faecalis will also alter its membrane fatty acid profile in response to changing temperatures, specifically increasing the proportion of unsaturated fatty acids in its membrane as the temperature is lowered (5). In contrast, in response to increasing temperature, species of Salmonella or Yersinia increase the proportion of cyclopropane fatty acids in their membranes (6, 7). While E. faecalis does produce cyclopropane fatty acids, it is not clear if they are required for acclimation to increasing temperatures.

Along with acclimating to changing temperatures, many bacteria will alter their membrane fatty acid composition in response to environmental acidification. As the pH lowers, Escherichia coli will increase the proportion of cyclopropane fatty acids in its membrane (8 and reviewed in reference 4). Deletion of the major cyclopropane synthase gene renders the organism acid sensitive (9). Other species, including the lactic acid-producing oral pathogens Streptococcus mutans and Streptococcus salivarius, will increase the percentage of unsaturated fatty acids found within their membranes as a result of environmental acidification (10–12). In the case of S. mutans, this increase is necessary for transmission of the pathogen between model host species as well as for full virulence (13).

In addition to modifying the membrane fatty acid content *de novo*, some bacterial species will utilize exogenously provided fatty acids from complex sources. We have documented that E. faecalis incorporates fatty acids from bile (commensal state) and serum (pathogenic state), thus altering its membrane composition (5). This in turn protected the organism from membrane-damaging agents such as the detergent sodium dodecyl sulfate (SDS) or the antibiotic daptomycin (5, 14), a lipopeptide that inserts into Gram-positive membranes in a calcium-dependent mechanism (15). Clinical isolates with genetic resistance to daptomycin have perturbations in the LiaFSR signaling pathway (16–19). Moreover, deletion of *liaR* (the response regulator) increases sensitivity to daptomycin (20, 21). Despite these results, we found that growth in fatty acid sources could protect even in the absence of a functional LiaFSR system, implying an alternative mechanism is responsible (14).

Complex fatty acid sources like serum can be utilized by some bacterial species, including E. faecalis, to overcome the need for *de novo* fatty acid biosynthesis. E. faecalis and other species, including Streptococcus agalactiae and Streptococcus pneumoniae, were able to grow in the presence of the fatty acid biosynthetic inhibitor cerulenin when supplied with human serum or other fatty acid sources (22, 23). However, this was not true of Staphylococcus aureus because of biochemical feedback in this species (23, 24). Regardless, when supplemented with individual exogenous fatty acids, the effects on bacterial growth are organism specific. For example, supplementation with palmitic acid (C_16:0_) was tolerated at high concentrations by S. aureus and B. subtilis yet inhibited growth of S. pneumoniae at far lower concentrations (25). In the same study, oleic acid (C_18:1 *cis*-9_) also was well-tolerated by S. aureus, whereas S. pneumoniae was highly sensitive to supplementation with this fatty acid.

As E. faecalis naturally resides in the mammalian intestine, where it is exposed to bile and is associated with surgical wound infections and exposed to serum, we predicted its tolerance to individual exogenous fatty acids would be distinct from that of the species described above and reflective of the composition of its native environments. In our previous study, we noted that E. faecalis could incorporate individual fatty acids into its membrane, and that the host-derived unsaturated fatty acids examined, oleic acid (C_18:1 *cis*-9_) and linoleic acid (C_18:2 *cis*-9,12_), provided protection from membrane-damaging agents (5). Based on these findings, we hypothesized that exogenous fatty acids do not produce the same physiological effect in E. faecalis and that the bacterium responds uniquely to each fatty acid provided. More specifically, we hypothesized that supplementation with host-derived unsaturated fatty acids, and not saturated fatty acids, would enhance growth in the presence of a fatty acid biosynthesis inhibitor and provide protection from membrane stress. Here, we demonstrate that individual fatty acids can severely impact cellular growth and morphology and that induction of membrane protection is specific to the fatty acid supplied and resulting membrane composition.

## RESULTS

### A membrane dominated by a single saturated fatty acid negatively impacts growth of E. faecalis.

**FIG 1:**
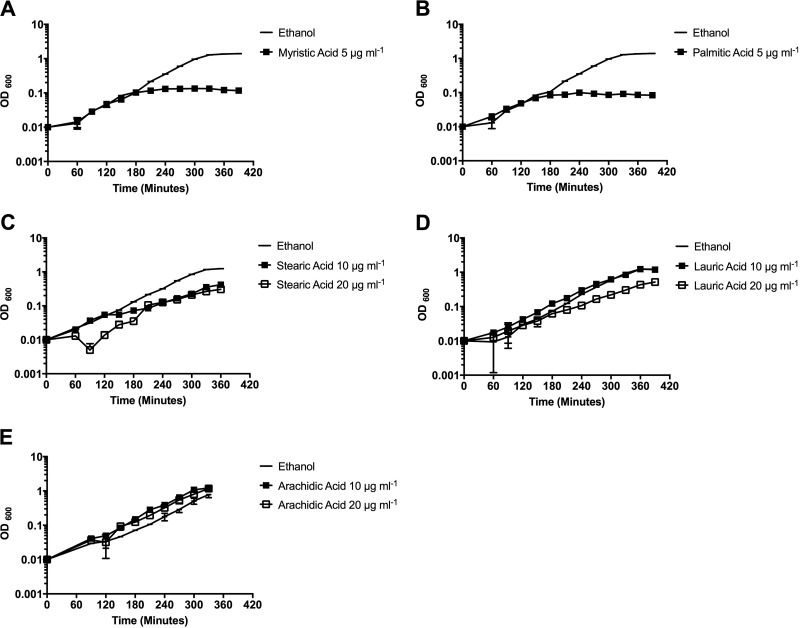
Long-term fatty acid growth of E. faecalis OG1RF in individual saturated fatty acid supplements. Overnight cultures of OG1RF were diluted to a final OD_600_ of 0.01 in BHI medium supplemented with either ethanol at a final concentration of 0.1% (solvent control) or the indicated fatty acid. (A) C_14:0_, myristic acid, 5 μg ml^−1^; (B) C_16:0_, palmitic acid, 5 μg ml^−1^; (C) C_18:0_, stearic acid, 10 μg ml^−1^, 20 μg ml^−1^; (D) C_12:0_, lauric acid, 10 μg ml^−1^, 20 μg ml^−1^; (E) C_20:0_, arachidic acid, 10 μg ml^−1^, 20 μg ml^−1^.

In our previous studies, we observed that when E. faecalis OG1RF was grown in rich medium, saturated fatty acids comprised approximately 50% of the total membrane fatty acid composition (5, 14). Indeed, growth in the presence of bile increased the saturated-to-unsaturated membrane fatty acid ratio, and these cells subsequently were protected from membrane-damaging agents (5). However, growth with individual saturated fatty acids did not offer the same protection (5). This implied that a simple increase in the saturated/unsaturated ratio was not responsible for protection from membrane damage and that E. faecalis was responding independently to different fatty acid supplements. To better resolve these findings, we examined growth of E. faecalis in the presence of saturated fatty acids of various lengths to discern their overall impact on cellular physiology (5, 14). We chose to examine saturated fatty acids 12 to 18 carbons in length, as these have been found in the membrane of E. faecalis, and several are also found in bile and serum. As the concentration of fatty acids within host fluids can be variable across studies, we analyzed the effects of 10 μg ml^−1^ on growth, as such concentrations are within the ranges reported in bodily fluids as well as in published analyses performed with other Gram-positive bacterial species (23, 26). Interestingly, supplementation with myristic acid (C_14:0_), palmitic acid (C_16:0_), and stearic acid (C_18:0_) all had negative impacts on growth rates (Fig. 1A, B, and C and Fig. S1A in the supplemental material). However, for stearic acid, although the generation time was less than that of the solvent control, the cells did continuously grow, even at concentrations as high as 20 μg ml^−1^ (Fig. 1C).

As growth in the presence of either myristic acid or palmitic acid was so poor (Fig. S1A), we reduced the concentration of supplement to 5 μg ml^−1^ and still observed negative impacts on growth (Fig. 1A and B). As shown, cells diluted in medium containing either fatty acid initially divided but then exhibited a prolong period of stasis lasting 18 h or more (Fig. 1A and B and Fig. S1A and B). We hypothesized that this outgrowth was due to the selection for a genetic mutation(s) within the population. To better address this, cultures on day 1 were supplemented with myristic acid (C_14:0_), palmitic acid (C_16:0_), or ethanol (solvent control) and grown for 24 h until late stationary phase. These cells then were diluted into fresh medium without fatty acid supplements and incubated for another 24 h (day 2). On day 3, cells were reinoculated into medium containing either myristic acid (C_14:0_) or palmitic acid (C_16:0_). As shown, these cells grew readily and consistently in the presence of fatty acid supplementation (Fig. S1C). These results indicate that the cells were no longer sensitive to these fatty acids and that this insensitivity was likely due to genetic mutation(s). Importantly, growth of cells with 20 μg ml^−1^ of stearic acid was not due to genetic mutation, as indicated in Fig. S2A.

Interestingly, not all saturated fatty acids examined had such negative growth consequences. Supplementation with either lauric acid (C_12:0_) or arachidic acid (C_20:0_) had little to no impact on cellular growth, even when provided at a final concentration of 20 μg ml^−1^ (Fig. 1D and E).

We hypothesized that these differences in growth could be attributed to the level of incorporation of the individual fatty acids within the cellular membrane. Consequently, total membrane fatty acid analysis via gas chromatography-fatty acid methyl ester (GC-FAME) was performed to compare the membrane profiles. As shown in Table 1, supplementation with myristic acid (C_14:0_), palmitic acid (C_16:0_), or stearic acid (C_18:0_) dominated the membrane profile, with C_14:0_ or C_16:0_ comprising over 80% of the total fatty acid content when supplemented, while C_18:0_ was over 50% of the total membrane content when stearic acid was provided in the culture medium. Cells supplemented with these fatty acids had statistically significantly reduced levels of the normally dominant unsaturated fatty acid, C_18:1 *cis*-11_, compared to those of the solvent control (*P* < 0.003). In all cases, the saturated/unsaturated fatty acid content ratio was increased compared to those of control cultures.

**TABLE 1:** E. faecalis OG1RF membrane fatty acid composition during exponential-phase growth^*a*^

Supplement	EtOH^*b*^	C_12:0_ (lauric acid)	C_14:0_^*c*^ (myristic acid)	C_16:1_ *_cis_*_-9_ (palmitoleic acid)	C_16:0_^*c*^ (palmitic acid)	C_18:1_ *_cis_*_-11_ (*cis*-vaccenic acid)	C_18:1_ *_cis_*_-9_ (oleic acid)	C_18:0_ (stearic acid)	C_18:2_ *_cis_*_-9,12_ (linoleic acid)	C_20:0_ (arachidic acid)
C_12:0_	0.87 ± 0.01	18.06 ± 3.30	ND	0.65 ± 0.12	0.81 ± 0.16	0.46 ± 0.05	1.07 ± 0.65	0.55 ± 0.06	1.08 ± 0.19	0.77 ± 0.101
C_14:0_	4.45 ± 0.14	4.72 ± 0.44	80.88 ± 0.54	1.93 ± 0.37	2.02 ± 0.04	0.53 ± 0.06	0.67 ± 0.075	3.53 ± 0.39	4.14 ± 0.64	3.56 ± 0.26
C_16:1_	6.22 ± 0.26	5.81 ± 0.94	1.79 ± 0.08	61.68 ± 9.46	2.66 ± 0.1	1.03 ± 0.25	1.37 ± 0.12	4.46 ± 0.27	4.62 ± 0.36	4.31 ± 0.36
C_16:0_	39.82 ± 0.29	37.00 ± 1.12	12.59 ± 0.60	13.05 ± 5.00	83.07 ± 0.65	2.33 ± 0.25	2.23 ± 0.42	17.02 ± 1.13	12.95 ± 2.59	37.57 ± 0.47
C_17.1_	ND	0.29 ± 0.50	ND	0.12 ± 0.21	ND	0.38 ± 0.13	ND	0.15 ± 0.13	ND	1.47 ± 0.23
C_17:0 2OH_	3.45 ± 0.26	0.46 ± 0.79	0.06 ± 0.07	1.9 ± 0.4	ND	1.65 ± 0.73	ND	0.9 ± 0.31	1.01 ± 0.15	4.41 ± 0.44
C_18:1_ *_cis_*_-9_	ND	1.81 ± 0.61	0.62 ± 0.03	0.43 ± 0.06	1.15 ± 0.08	ND	84.52 ± 0.61	ND	ND	ND
C_18:1_ *_cis_*_-11_	39.05 ± 0.42	25.52 ± 4.31	2.43 ± 0.21	15.6.9 ± 2.02	6.98 ± 0.16	91.28 ± 0.66	ND	18.96 ± 1.88	4.71 ± 0.46	29.98 ± 1.16
C_18:0_	5.48 ± 0.27	5.16 ± 0.12	1.42 ± 0.04	2.12 ± 1.9	2.62 ± 0.09	0.53 ± 0.09	0.79 ± 0.23	53.41 ± 3.05	2.22 ± 0.54	6.33 ± 0.44
C_20:1_	ND	ND	ND	0.08 ± 0.14	ND	ND	ND	0.32 ± 0.072	ND	0.27 ± 0.25
C_20:0_	ND	ND	ND	ND	ND	ND	7.9 ± 1.08	ND	ND	10.78 ± 1.16
C_18:2_	ND	ND	ND	0.73 ± 0.13	ND	ND	ND	ND	68.69 ± 4.05	ND
C_19 cyclo_	0.65 ± 0.44	0.31 ± 0.53	ND	1.87 ± 0.1	ND	1.73 ± 0.14	1.45 ± 0.16	0.54 ± 0.05	0.47 ± 0.27	0.36 ± 0.31
Others^*d*^	ND	0.88 ± 1.51	0.11 ± 0.19	1.84 ± 0.5	0.67 ± 0.6	0.08 ± 0.12	ND	0.167 ± 0.28	0.29 ± 0.5	0.19 ± 0.311
Sat/Unsat^*e*^	1.12 ± 0.02	1.98 ± 0.44	19.6 ± 0.75	0.23 ± 0.11	8.21 ± 0.13	0.04 ± 0.004	0.041 ± 0.004	3.13 ± 0.35	0.26 ± 0.061	1.64 ± 0.096
(C_10_-C_17_)/(C_18_-C_20_)^*f*^	1.21 ± 0.01	2.06 ± 0.44	20.9 ± 0.43	3.97 ± 0.92	8.23 ± 0.23	0.068 ± 0.007	0.057 ± 0.006	0.36 ± 0.02	0.32 ± 0.066	1.09 ± 0.46

a Percentage of total membrane content as determined by GC-FAME by Microbial ID, Inc.; shown are averages ± standard deviations from three independent experiments. Overnight cultures were diluted in medium supplemented with the indicated fatty acids to a final concentration of 10 μg ml^−1^ and grown until mid-exponential phase. ND, not determined.

b Ethanol was added to a final concentration of 0.1%.

c Due to poor growth, these cells were harvested after 6 h of growth for analysis.

d Others indicates the total of all fatty acids comprising <1% of the total membrane content.

e Total fatty acid saturation ratios.

f Total fatty acid length ratios, including both saturated and unsaturated fatty acids.

Although the above-described fatty acids had measureable impacts on generation times and major consequences on membrane fatty acid composition, there was little impact on growth rates in the presence of lauric (C_12:0_) or arachidic (C_20:0_) acid (Fig. 1D and E) and the total membrane composition (Table 1). The only major alterations on fatty acid composition were the consistent, detectable amounts of these fatty acids in the membrane upon supplementation (18% for C_12:0_, 11% for C_20:0_) and a statistically significant but milder reduction in C_18:1 *cis*-11_ compared to levels of other examined saturated fatty acids.

**FIG 2:**
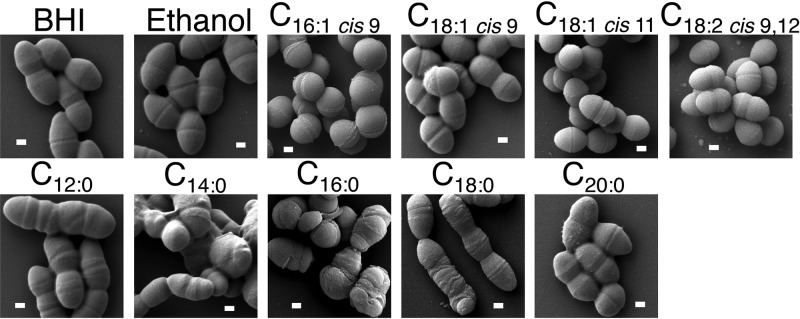
Scanning electron microscope images of E. faecalis during long-term growth with fatty acid supplements. Fatty acids were added to a final concentration of 10 μg ml^−1^ unless indicated otherwise. (Top row, left to right) OG1RF grown in BHI alone, ethanol control (0.1% final concentration), palmitoleic acid, *cis*-vaccenic acid, oleic acid, linoleic acid. (Bottom row, left to right) OG1RF grown in the presence of lauric acid, myristic acid (final concentration, 5 μg ml^−1^), palmitic acid (final concentration, 5 μg ml^−1^), stearic acid, arachidic acid. White bars represent 200 nm. Shown are representative images from *n* = 3 experiments (see Materials and Methods).

Overall, alterations in cellular morphology mimicked the negative impacts on growth observed with supplementation of specific saturated fatty acids. The more toxic an exogenously supplied fatty acid was to cellular growth, the more altered the cellular morphology. As shown in Fig. 2, OG1RF exhibits a lancet shape, and cells are typically found as pairs (diplococci). Growth with toxic fatty acids, such as myristic or palmitic acids, led to severe cellular distortion, with the placement of cell septa aberrant or absent in many cases. Overall, cell size and shape were very inconsistent across the population, with some cells appearing small and round and others rather elongated. While cells were able to grow in the presence of stearic acid (C_18:0_), we did observe an alteration in cellular morphology in a portion of the population (Fig. 2 and the zoomed-out image in Fig. S3A). Exogenously supplied lauric acid and arachidic acid had relatively negligible impacts on growth (Fig. 1D and E), and this too is reflective in the less severe alterations in cellular morphology (Fig. 2). We did note that cells supplemented with lauric acid were more elongated and found in chains four cells in length, with some harboring misplaced septa, unlike control cells. However, no impacts were observed with C_20:0_, which was also incorporated least (Table 1); thus, negative impacts on cellular growth via saturated fatty acids may be due the amount of incorporation (see Discussion).

### Supplementation with unsaturated fatty acids can significantly alter the membrane composition of E. faecalis but are not toxic.

Previous studies indicated that growth in either bile or human serum led to incorporation of host-derived unsaturated fatty acids and subsequent tolerance to the membrane-targeting antibiotic daptomycin (5, 14). However, we did not conclude if this response was limited to host-derived unsaturated fatty acids or was a generic response to supplementation with any unsaturated fatty acid. To deduce whether E. faecalis also responds uniquely to host-derived unsaturated fatty acids, we examined growth effects upon supplying either native unsaturated fatty acids or host-associated fatty acids.

**FIG 3:**
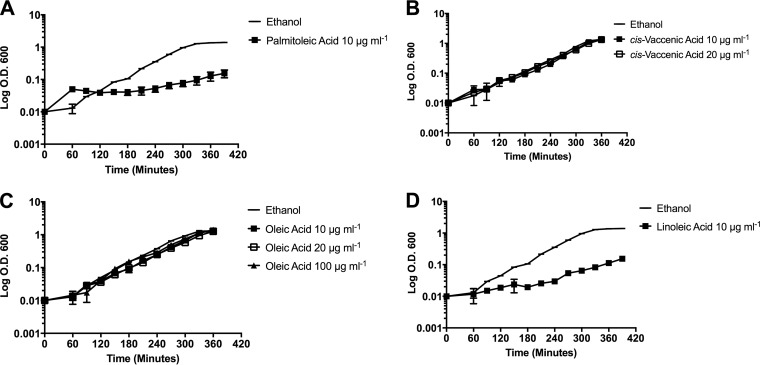
Long-term fatty acid growth of E. faecalis OG1RF in individual unsaturated fatty acid supplements. Cultures were grown as described for Fig. 1 with either ethanol at a final concentration of 0.1% (solvent control) or the indicated fatty acid. (A) C_16:1 *cis*-9_, palmitoleic acid, 10 μg ml^−1^; (B) C_18:1 *cis*-11_, *cis*-vaccenic acid, 10 μg ml^−1^, 20 μg ml^−1^; (C) C_18:1 *cis*-9_, oleic acid, 10 μg ml^−1^, 20 μg ml^−1^, 100 μg ml^−1^; (D) C_18:2 *cis*-9,12_, linoleic acid, 10 μg ml^−1^.

Unlike what we observed for the saturated fatty acid counterparts, unsaturated C_16_ and C_18_ fatty acids were not toxic for growth. Although supplementation with palmitoleic acid (C_16:1 *cis*-9_, a native member of the membrane) (Table 1) or linoleic acid (C_18:2 *cis*-9,12_, a nonnative fatty acid found in host fluids) did result in reduced growth rates compared to those of control cultures, especially at higher concentrations, the cultures grew continuously (Fig. 3A and D and Fig. S2B). These growth rates remained unchanged upon reexposure to the fatty acid (Fig. S2A), indicating that outgrowth was not due to a genetically resistant subpopulation, unlike what was observed for palmitic acid or myristic acid supplementation.

Examination of two additional C_18_ unsaturated fatty acids, the native enterococcal fatty acid *cis*-vaccenic acid (C_18:1 *cis*-11_) and the nonnative fatty acid oleic acid (C_18:1 *cis*-9_), had little to no impact on generation time or growth rate, even when provided at higher concentrations (Fig. 3B and C). Thus, while the unsaturated fatty acids examined here may impact growth rates, they were not toxic for growth and did not lead to the selection of genetic suppressors.

We wanted to determine whether the milder growth phenotypes associated with unsaturated fatty acid supplementation were linked to decreased incorporation into the membrane. As shown in Table 1, all unsaturated fatty acids examined, whether they are found natively within the membrane of OG1RF or not, comprised the majority of the total fatty acid composition. There was a concomitant decrease in the proportion of saturated fatty acids within the membrane, in particular a decrease in the proportion of C_16:0_, which comprised over 37% of the total content in control cultures. With the exception of supplementation with *cis*-vaccenic acid, the amount of the dominant unsaturated fatty acid, C_18:1 *cis*-11_, was also greatly reduced compared to that of control cells. Surprisingly, only supplementation with oleic acid (C_18:1 *cis*-9_) led to the detection of significant amounts of C_20:0_ within the membrane. This fatty acid normally is not detected in E. faecalis, suggesting that this is a specific consequence of growth with oleic acid (Table 1).

We also examined cellular morphology, as alterations in cellular shape are often reflected in decreased generation time. As shown in Fig. 2, overall morphology was not altered upon growth in any of the unsaturated C_18_ fatty acids examined. However, cells grown with exogenous palmitoleic acid (C_16:1 *cis*-9_) did not have the typical lancet morphology but instead appeared more rounded or domed, yet the cell septum appeared to be at the midpoint for the diplococci, and the morphology was consistent across biological replicates and multiple fields examined (see Materials and Methods and Fig. S3B). Thus, despite their high incorporation in the enterococcal membrane, the unsaturated fatty acids examined here were fairly innocuous in terms of impacting growth rate and morphology.

### Unsaturated fatty acids can override the need for *de novo* fatty acid biosynthesis.

Thus far, our data support the hypothesis that E. faecalis does not possess a generic response to exogenously supplied fatty acids but instead responds to each individually. For some bacterial species, supplementation with fatty acids can override the need for *de novo* membrane fatty acid biosynthesis (22, 23). These species can generate their membranes entirely from environmental sources, rendering them resistant to the inhibitor cerulenin, which prevents elongation of fatty acids during biosynthesis. Indeed, previous studies demonstrated that E. faecalis could overcome cerulenin inhibition when grown in the presence of serum (22). We wanted to conclude which fatty acids could be used to overcome inhibition by cerulenin so that we could better discern the varied, inherent physiological responses of E. faecalis to fatty acids.

**FIG 4:**
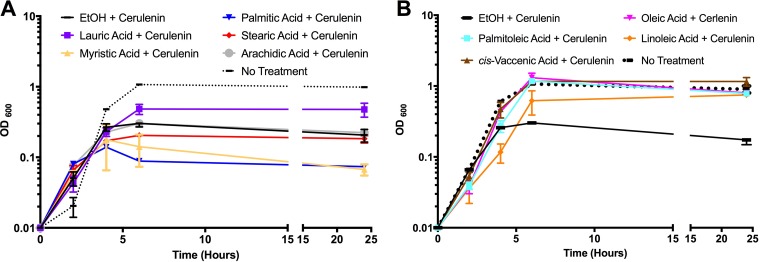
Supplementation with unsaturated fatty acids, but not saturated fatty acids, can overcome growth inhibition by cerulenin. Cells were grown as described for Fig. 1 and diluted back into medium containing cerulenin at 5 μg ml^−1^ and either ethanol at 0.1% final concentration or the indicated fatty acid at 5 μg ml^−1^. As a control, cells were also diluted back into medium lacking the inhibitor or fatty acids and indicated as no treatment. (A) Saturated fatty acid supplements; (B) unsaturated fatty acid supplements. Shown are the averages and standard deviations from *n* = 3 experiments.

As noted above, since supplementation with specific, individual fatty acids could be toxic for growth, we elected to test each supplement at a final concentration of 5 μg ml^−1^. For the saturated fatty acids tested, no single fatty acid conferred growth in the presence of cerulenin (Fig. 4A). We did note that cells supplemented with lauric acid (C_12:0_) reached a higher initial optical density before entering stasis and retained an elevated optical density compared to that of other supplements even 24 h after treatment. It did not, however, reach the same optical density as the no-cerulenin treatment control (Fig. 4A). As the concentration of lauric acid (5 μg ml^−1^) may not have been sufficient to restore growth, we increased the concentration (10 μg ml^−1^) and repeated the analysis. Again, cells did not reach the optical density of the no-cerulenin control (Fig. S4), confirming that E. faecalis cannot utilize lauric acid to overcome cerulenin inhibition.

Unlike supplementation with the indicated saturated fatty acids, OG1RF was able to overcome cerulenin-induced growth inhibition when supplemented with any of the unsaturated fatty acids examined here (Fig. 4B). Thus, E. faecalis can utilize unsaturated fatty acids to overcome fatty acid biosynthesis inhibition, and a membrane comprised solely of unsaturated fatty acids is not lethal to the organism.

### Protection from membrane damage: specific to host-derived unsaturated fatty acids.

We have demonstrated that when E. faecalis is diluted into medium containing bovine bile, human serum, or oleic acid, grown to exponential phase (here referred to as long-term fatty acid growth) and then exposed to membrane-damaging agents, there is an increased number of survivors compared to cells grown without supplementation (5, 14). Could any fatty acid then induce protection from membrane-damaging agents, i.e., do all fatty acids induce the same physiologic response? Given the inherent toxicity of some saturated fatty acids during long-term fatty acid growth, measuring protective responses was challenging. We elected to perform a short-term fatty acid exposure and then measure sensitivity to the membrane-damaging agent daptomycin to avoid the potential toxic effects of long-term fatty acid exposure.

Before examining membrane protection after short-term fatty acid exposure, we first determined if fatty acids could be incorporated in a shortened time frame. For these analyses, we focused on those fatty acids that altered the membrane most significantly during long-term fatty acid growth (Table 1). OG1RF was initially grown in liquid medium until mid-logarithmic phase (optical density at 600 nm [OD_600_] of ∼0.3), and then fatty acids or solvent control was added to the growth medium for 30 min (referred to as short-term fatty acid growth; see Materials and Methods). We noted that the cultures continued to grow for at least 30 min after fatty acid addition (Fig. S5). All of the fatty acids examined were incorporated, as reflected in their altered membrane profiles (Table 2), although there were some notable differences in the amount of incorporation of each fatty acid. As shown in Table 2, short exposure of either palmitic (C_16:0_) or stearic (C_18:0_) acid led to a statistically significant reduction in the amount of the dominant unsaturated fatty acid (C_18:1 *cis*-11_) within the membrane (*P* < 0.005 and *P* < 0.05, respectively). Short-term exposure to either of these fatty acids greatly increased the saturated/unsaturated fatty acid ratio compared to that of control cultures (*P* < 0.005).

**TABLE 2:** E. faecalis OG1RF membrane fatty acid composition following fatty acid spike-in^*a*^

Supplement	*T*_0_	EtOH^*b*^	C_16:1_ *_cis_*_-9_ (palmitoleic acid)	C_16:0_ (palmitic acid)	C_18:1_ *_cis_*_-11_ (*cis*-vaccenic acid)	C_18:1_ *_cis_*_-9_ (oleic acid)	C_18:0_ (stearic acid)	C_18:2_ *_cis_*_-9,12_ (linoleic acid)
C_12:0_	0.82 ± 0.03	0.58 ± 0.02	0.78 ± 0.11	0.31 ± 0.03	0.51 ± 0.05	0.57 ± 0.04	0.44 ± 0.03	1.04 ± 0.14
C_14:0_	4.34 ± 0.17	3.48 ± 0.05	3.53 ± 0.13	1.88 ± 0.04	2.58 ± 0.02	3.22 ± 0.06	3.12 ± 0.16	3.46 ± 0.04
C_16:1_	6.9 ± 0.34	5.47 ± 0.19	15.16 ± 0.31	2.81 ± 0.08	3.53 ± 0.03	4.07 ± 0.06	4.23 ± 0.26	5.07 ± 0.13
C_16:0_	35.76 ± 0.37	35.20 ± 0.29	32.13 ± 0.22	62.95 ± 0.87	24.24 ± 0.86	25.99 ± 0.71	24.16 ± 0.10	28.96 ± 0.4
C_17.1_	0.86 ± 0.05	1.1 ± 0.06	0.96 ± 0.02	0.51 ± 0.06	0.78 ± 0.03	0.88 ± 0.04	0.73 ± 0.04	0.6 ± 0.03
C_17:0 2OH_	3.52 ± 0.1	5.52 ± 0.36	3.82 ± 0.27	2.82 ± 0.32	3.96 ± 0.25	3.84 ± 0.19	3.94 ± 0.14	1.99 ± 0.26
C_18:1_ *_cis_*_-9_	ND	ND	ND	ND	ND	13.83 ± 1.93	ND	ND
C_18:1_ *_cis_*_-11_	41.43 ± 0.21	40.9 ± 0.71	36.06 ± 0.61	22.45 ± 0.84	59.25 ± 1.16	26.74 ± 0.5	25.01 ± 0.45	27.59 ± 0.81
C_18:0_	4.96 ± 0.21	6.12 ± 0.08	6.24 ± 0.31	5.08 ± 0.04	3.57 ± 0.17	3.83 ± 0.13	37.12 ± 0.56	3.97 ± 0.06
C_20:1_	0.35 ± 0.1	0.38 ± 0.02	0.32 ± 0.03	0.28 ± 0.02	0.27 ± 0.04	0.26 ± 0.05	0.23 ± 0.02	ND
C_20:0_	ND	ND	ND	ND	0.05 ± 0.09	15.3 ± 1.91	ND	ND
C_18:2_	ND	ND	ND	ND	ND	ND	ND	26.375 ± 1.29
C_19 cyclo_	0.62 ± 0.40	0.31 ± 0.53	0.87 ± 0.02	0.58 ± 0.03	1.03 ± 0.01	1.06 ± 0.04	0.69 ± 0.02	0.82 ± 0.13
Others^*c*^	0.43 ± 0.91	0.28 ± 0.28	0.13 ± 0.22	0.32 ± 0.1	0.22 ± 0.03	0.4 ± 0.1	0.33 ± 0.20	0.14 ± 0.23
Sat/Unsat^*d*^	0.93 ± 0.02	0.95 ± 0.02	0.81 ± 0.0.2	2.7 ± 0.13	0.49 ± 0.02	1.07 ± 0.09	2.15 ± 0.06	0.63 ± 0.001
(C_10_-C_17_)/(C_18_-C_20_)^*e*^	1.11 ± 0.01	1.06 ± 0.02	1.3 ± 0.03	2.51 ± 0.11	0.56 ± 0.03	0.63 ± 0.02	0.58 ± 0.01	0.7 ± 0.01

a Percentage of total membrane content as determined by GC-FAME by Microbial ID, Inc.; shown are averages ± standard deviations for three independent experiments. Cells were grown to mid-exponential phase (*T*_0_), and fatty acids were added to a final concentration of 10 μg ml^−1^ for 30 min and then harvested for GC-FAME.

b Ethanol was added to a final concentration of 0.1%.

c Others indicates the total of all fatty acids comprising <1% of the total membrane content.

d Total fatty acid saturation ratios.

e Total fatty acid length ratios, including both saturated and unsaturated fatty acids.

Short-term fatty acid growth with unsaturated fatty acids led to more variable membrane compositions, dependent upon the fatty acid examined. As seen in Table 2, supplementation with palmitoleic acid (C_16:1 *cis*-9_), *cis*-vaccenic acid (C_18:1 *cis*-11_), oleic acid (C_18:1 *cis*-9_), or linoleic acid (C_18:2 *cis*-9,12_) led to a decreased proportion of C_16:0_ found within the membrane and, overall, a decrease in the saturated/unsaturated fatty acid content ratio. The increase in the amount of individual unsaturated fatty acid varied across supplements with a 10% increase in C_16:1 *cis*-9_ (palmitoleic acid supplementation) to as high as 26% for C_18:2 *cis*-9,12_ (linoleic acid supplementation). This increase in the proportion of C_18:2 *cis*-9,12_ upon linoleic acid supplementation coincided with a significant decrease in the amount of C_18:1 *cis*-11_ (*cis*-vaccenic acid) found in the membrane (*P* < 0.05). The other major observation upon short-term fatty acid exposure was the appearance of C_20:0_ in the membranes of cells exposed to oleic acid (C_18:1 *cis*-9_). While C_18:1 *cis*-9_ comprised only 13% of the total content upon 30 min of exposure to oleic acid, C_20:0_ comprised 15% of the membrane content (*P* < 0.01) (Table 2). This increase in C_20:0_ was surprising given that this was even greater than what was observed for long-term exposure to oleic acid (Table 1).

**FIG 5:**
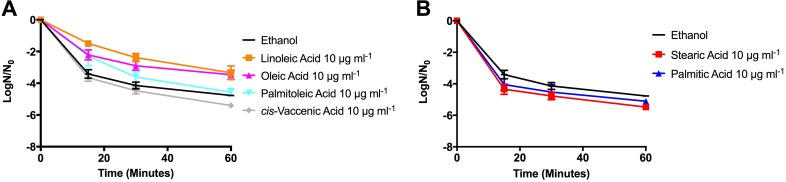
Short exposure to specific exogenously supplied fatty acids protects E. faecalis OG1RF from daptomycin challenge. (A) Unsaturated fatty acid supplementation and challenge with 30 μg ml^−1^ daptomycin. Both oleic acid and linoleic acid supplementation led to increased numbers of survivors compared to the control culture (*P* < 0.05) for all time points. Palmitoleic acid supplementation led to increased numbers of survivors compared to the control only at *T* = 15 min (*P* < 0.05). (B) Saturated fatty acid supplementation and challenge with 30 μg ml^−1^ daptomycin. Supplementation with stearic acid led to significantly fewer survivors (*P* < 0.05) than the control culture at all time points. For all samples, shown are the averages ± standard deviations for *n* = 3 experiments.

Since OG1RF could incorporate exogenous fatty acids within 30 min (Table 2) with minimal impact on growth within that time frame (Fig. S5), we examined if short-term fatty acid exposure could induce tolerance to the antibiotic daptomycin, thus avoiding potential toxicity from long-term fatty acid exposure. As shown in Fig. 5A, even though cultures treated with linoleic acid (C_18:2 *cis*-9,12_) or oleic acid (C_18:1 *cis*-9_) had very different saturated/unsaturated fatty acid ratios from each other (Table 2), both cultures had significantly improved survival to daptomycin treatment. However, *cis*-vaccenic acid exposure (C_18:1 *cis*-11_) did not induce daptomycin tolerance, even though it is the same length and unsaturated, like oleic acid and linoleic acid (see Discussion). Treatment with palmitoleic acid (C_16:1 *cis*-9_) induced protection only within the first 15 min of exposure. Thus, supplementation with unsaturated fatty acids is not equivalent in either the membrane profiles generated or how they contribute to daptomycin tolerance.

Given the varied protective responses observed for the unsaturated fatty acids supplied, we examined whether the saturated fatty acids also produced variable protective responses. As shown in Fig. 5B, supplementation with neither palmitic nor stearic acids could induce daptomycin tolerance. Regarding stearic acid supplementation, the C_10_-C_17_/C_18_-C_20_ ratio for those cells was 0.58, much closer to the ratio (0.63) produced by oleic acid (induced daptomycin tolerance) than that of the solvent control (0.95) (Table 2). Combined, these data imply that E. faecalis responds independently to environmental fatty acids, regardless of similarities in overall fatty acid tail length or membrane saturation. Consequently, we conclude that there is no common physiological response induced by individual fatty acids that leads to daptomycin tolerance.

## DISCUSSION

In this study, we examined the physiological effects on E. faecalis of exogenous saturated and unsaturated fatty acids that are produced natively by the organism and those found within the environmental niches of this commensal and hospital-acquired pathogen. We observed that E. faecalis does not have a generalized phenotypic response to exogenously supplied fatty acids. It responds uniquely to each fatty acid examined. This is particularly evident when examining growth of the organism with fatty acids 18 carbons in length. Growth with stearic acid, C_18:0_, led to altered morphology of the organism (Fig. 3), the inability to overcome inhibition by the fatty acid biosynthetic inhibitor cerulenin (Fig. 4), and the absence of daptomycin tolerance (Fig. 5). However, exogenously supplied *cis*-vaccenic acid (C_18:1 *cis*-11_), the dominant, native, unsaturated fatty acid, did not alter morphology and allowed growth in the presence of cerulenin. Supplementation with the nonnative unsaturated fatty acids oleic acid (C_18:1 *cis*-9_) and linoleic acid (C_18:2 *cis*-9,12_) restored growth in the presence of cerulenin (Fig. 4) and induced daptomycin tolerance (Fig. 5), yet linoleic acid supplementation significantly impaired generation time (Fig. 3D). Thus, despite being the same length and, in the case of oleic acid and *cis*-vaccenic acid, different only in the placement of the unsaturated fatty acid bond, E. faecalis had altered physiology when supplemented with different fatty acids 18 carbons in length. These results, along with other data generated in this work, support the hypothesis that E. faecalis responds independently to the fatty acids it encounters in its environment.

Not only did fatty acids have independent impacts on membrane composition and stress survival but they also generated a variety of morphologies. As noted, supplementation with saturated fatty acids had severe effects with cells having irregular shapes and misplacement of septa (Fig. 2). This occurred even with lauric acid (C_12:0_) supplementation, which had minimal impacts on generation time. Likely the altered membrane fatty acid content leads to misplacement of cellular divisional machinery. Studies have shown that altered phospholipid composition can impact localization of both divisional proteins and those involved in respiration (reviewed in reference 27), but studies examining bacterial fatty acid composition on cellular protein placement are lacking. It is surprising, though, that supplementation with stearic acid at a concentration that does not hinder cellular growth (10 μg ml^−1^) (Fig. 1) led to altered morphology (Fig. 2). Some cells did appear to have the more typical lancet shape (see Fig. S3A in the supplemental material); it may be that these cells have a membrane composition more similar to those grown without stearic acid, allowing growth to continue.

Further, supplementation with the unsaturated fatty acid palmitoleic acid resulted in cells having a distinct rounded or domed morphology. Given this altered cellular appearance and the reduction in generation time upon growth in palmitoleic acid, it is possible that this membrane composition impacts proper localization of divisional machinery. This domed morphology of cultures grown with palmitoleic acid was consistent across the population (Fig. S3B), whereas cells grown with the equivalent saturated fatty acid, palmitic acid, were far more inconsistent in shape and size (Fig. 2). Thus, differences in saturation, despite equivalent tail lengths, can have altered impact on cellular morphology.

As acknowledged, it is unclear how membrane fatty acids influence membrane protein localization, abundance, and activity within OG1RF. Studies in S. mutans, an oral bacterium, have showed that cells grown in the presence of exogenous fatty acids as well as a strain deleted for specific membrane fatty acids had altered membrane protein activities and abundances that could contribute to cellular growth and physiology (28, 29). Further, membrane fatty acids are attached to a polar head group within a membrane; supplying exogenous fatty acids may alter the polar head group composition in E. faecalis. Phospholipid changes in turn could contribute to alterations in the protein lipid ratio or, again, the localization and activities of numerous membrane proteins (18, 27, 30–35).

For saturated fatty acid supplementation, the effects on OG1RF physiology were correlated with the levels of the exogenous fatty acid found within the membrane. In particular, C_12:0_ and C_20:0_ had only minor effects on the bacterium, and they also were incorporated at lower levels in the membrane than other saturated fatty acids examined (Table 1). Why are these fatty acids so poorly incorporated? Although not confirmed, E. faecalis likely incorporates exogenous fatty acids in the same manner as S. aureus (36, 37). In S. aureus, fatty acids are thought to passively flip into the cytoplasmic membrane, where they are bound to FakB proteins that act along with FakA, resulting in phosphorylation of the fatty acid. Upon phosphorylation, the acyl chain can be used in phospholipid synthesis or the phosphate can be exchanged for the acyl carrier protein (ACP) and enter the fatty acid elongation cycle. In studies with S. aureus, Parsons et al. demonstrated that C_12:0_ was a poor substrate for binding to either of the two FakB proteins found within that organism (36). Perhaps C_12:0_ is also a poor substrate for the FakB homologs of E. faecalis and consequently are not available for use by the cell. Alternatively, C_12:0_ may be readily routed to the fatty acid elongation cycle and thus does not accumulate to high levels within the membrane. Additional studies to examine the activities of the FakB homologs in E. faecalis are needed to better resolve these possibilities. For C_20:0_ supplementation, there were no membrane fatty acids detected longer than that within the membrane, and neither have any of our past growth conditions indicated that E. faecalis has fatty acid tails longer than this in its membrane (5, 14). We hypothesize that C_20:0_ is a poor substrate for the Fak system, which is likely the reason for minimal incorporation and effects on the cell. Future experiments are geared to test this hypothesis.

The saturated fatty acids incorporated at high levels, myristic acid (C_14:0_) and palmitic acid (C_16:0_), likely are excellent substrates for the enterococcal Fak system. This was shown to be the case for S. aureus (36). This high incorporation level then likely inadvertently contributes to cellular toxicity: clearly, a membrane composed primarily of saturated, shorter-chained fatty acids is not conducive to the proper cellular division of E. faecalis. Also, as mentioned above, the fatty acid composition likely is impacting the abundance and activities of membrane proteins. Current genomic analyses are being performed to determine whether the outgrowth mutations are associated with the Fak system or another pathway.

One additional observation made above is that the length of the fatty acid tail does not dictate physiology. *Cis*-vaccenic acid, when provided exogenously, is well tolerated and even allows E. faecalis to overcome cerulenin inhibition, and it did not induce daptomycin tolerance; only eukaryote-derived unsaturated C_18_ fatty acids did. This may be due to differences in downstream consequences on membrane proteins, the phospholipid content, or other cellular or biochemical processes. Ongoing experiments are geared to better resolve the effects of different C_18_ fatty acids on the physiology of E. faecalis.

Individual fatty acid supplementation had a wide array of growth consequences on the organism, but for those examined, unsaturated fatty acids were less toxic than the saturated fatty acids. Further, the length of the saturated fatty acid tail was critical in determining toxicity, as noted with myristic (C_14:0_) and palmitic (C_16:0_) acids having the most severe consequences on growth. The toxicity of saturated fatty acids could be because of a reduction of membrane fluidity (38). The extreme sensitivity to myristic and palmitic acid may be because the membrane is not only more rigid but also more compressed, which could impact proper functioning of membrane processes. On the other hand, a membrane comprised of unsaturated fatty acids clearly was not too fluid for growth (Fig. 3 and 4). The increased fluidity due to incorporation of these unsaturated fatty acids may be more easily compensated by alterations in the phospholipid content and/or membrane protein composition. Further analyses are warranted to elucidate these mechanisms.

Overall, we have demonstrated that exogenous fatty acids do not induce a common physiological response in E. faecalis. The organism responds independently to each fatty acid it encounters. Further, eukaryote-derived unsaturated fatty acids clearly trigger unique responses that better protect the organism from membrane damage.

## MATERIALS AND METHODS

### Bacterial strains and growth conditions.

E. faecalis OG1RF was grown statically in brain heart infusion medium (BHI; BD Difco) at 37°C. For long-term fatty acid exposure, overnight cultures were diluted into fresh medium to an optical density at 600 nm (OD_600_) of 0.01 with supplements added to the final concentration as indicated above. For short-term fatty acid exposure, overnight cultures were diluted as described above into BHI medium and grown until an OD_600_ of ∼0.2 to 0.3 when supplements were then added, as indicated above. All fatty acids and chemicals were purchased from Sigma-Aldrich unless noted otherwise.

### GC-FAME preparation and analysis.

Cells were grown to log phase with the indicated supplements as noted above. Cells (15-ml aliquots) were flooded with 30 ml of 1× phosphate-buffered saline (PBS), pelleted, and then washed twice with 15 ml 1× PBS and stored at −80°C prior to shipment to Microbial ID, Inc. (Newark, DE). Cells were subjected to saponification with a sodium hydroxide-methanol mixture, a methylation step, and a hexane extraction prior to GC-FAME analysis (39).

### SEM preparation and analysis.

Overnight cultures of E. faecalis OG1RF were diluted as described above in medium containing 10 μg ml^−1^ of the fatty acid or solvent control. Initial sample preparation and fixations for scanning electron microscopy (SEM) were previously described (5), with the noted modifications. Following osmium tetroxide fixation, SEM samples were added to 5- by 5-mm silicon chips (Ted Pella, Inc.). Silicon chips were passed through a graded ethanol series (25, 50, 70, 95, and 100%) for 15 min at each interval. Chips then were placed in a LADD critical point dryer for three 10-min cycles. Each silicon chip was coated with gold for 10 s prior to visualization. Samples were viewed using a Zeiss Auriga 40 at the Center for Advanced Microscopy and Imaging at the University of Tennessee at a kEV of 1.0. Biological triplicates were performed for each growth condition, with a minimum of 10 fields examined per replicate.

### Membrane challenge assays.

Cells were grown in BHI medium until exponential phase (OD_600_ of ∼0.25 to 0.3) and then exposed to 10 μg ml^−1^ of stearic acid (C_18:0_), palmitic acid (C_16:0_), linoleic acid (C_18:2 *cis*-9,12_), oleic acid (C_18:1 *cis*-9_), *cis*-vaccenic acid (C_18:1 *cis*-11_), palmitoleic acid (C_16:1 *cis*-9_), or the solvent control ethanol (0.1% final volume) for 30 min. Aliquots (10 ml) were flooded with 20 ml of 1× PBS, subjected to vacuum filtration, and washed and filtered two more times with 20 ml of 1× PBS. After washing, cells were resuspended in BHI medium containing 1.4 mM CaCl_2_ and treated with 30 μg ml^−1^ of daptomycin. Serial dilutions were plated onto BHI agar at 0, 15, 30, and 60 min after exposure to daptomycin. The log ratio of survivors over time was calculated for three biological replicates, and shown are the averages and standard deviations for each experiment.

### Statistical analysis.

Differences in the fatty acid content between growth conditions as well as differences in the log ratio of survivors over time were determined using two-tailed, unpaired Student's *t* tests as indicated in the text.

## Supplementary Material

Supplemental material
