# Supplementary material for: Enterococcus faecalis Responds to Individual Exogenous Fatty Acids Independently of Their Degree of Saturation or Chain Length
Source: Appl Environ Microbiol. 2017 Dec 15;84(1):e01633-17. doi: 10.1128/AEM.01633-17 (PMC5734047; doi:10.1128/AEM.01633-17)
Supplement: Supplemental material [file supp_84_1_e01633-17__index.html]

Enterococcus faecalis Responds to Individual Exogenous Fatty Acids Independently of Their Degree of Saturation or Chain Length — Supplemental material 

# Enterococcus faecalis Responds to Individual Exogenous Fatty Acids Independently of Their Degree of Saturation or Chain Length

## Supplemental material

- Supplemental file 1 -

  Growth of OG1RF with myristic acid or palmitic acid (Fig. S1); growth of OG1RF with stearic acid, linoleic acid, or palmitoleic acid (Fig. S2); scanning electron microscope images of *E. faecalis* during long-term growth with fatty acid supplements (Fig. S3); OG1RF cannot overcome cerulenin inhibition when provided with lauric acid (Fig. S4); growth of OG1RF following fatty acid addition during exponential phase (Fig. S5).

  PDF, 5.5M
