# Supplementary material for: Enterococcus faecalis Responds to Individual Exogenous Fatty Acids Independently of Their Degree of Saturation or Chain Length
Source: Appl Environ Microbiol. 2017 Dec 15;84(1):e01633-17. doi: 10.1128/AEM.01633-17 (PMC5734047; doi:10.1128/AEM.01633-17)
Supplement: Supplemental material [file AEM.01633-17_zam001188230s1.pdf]

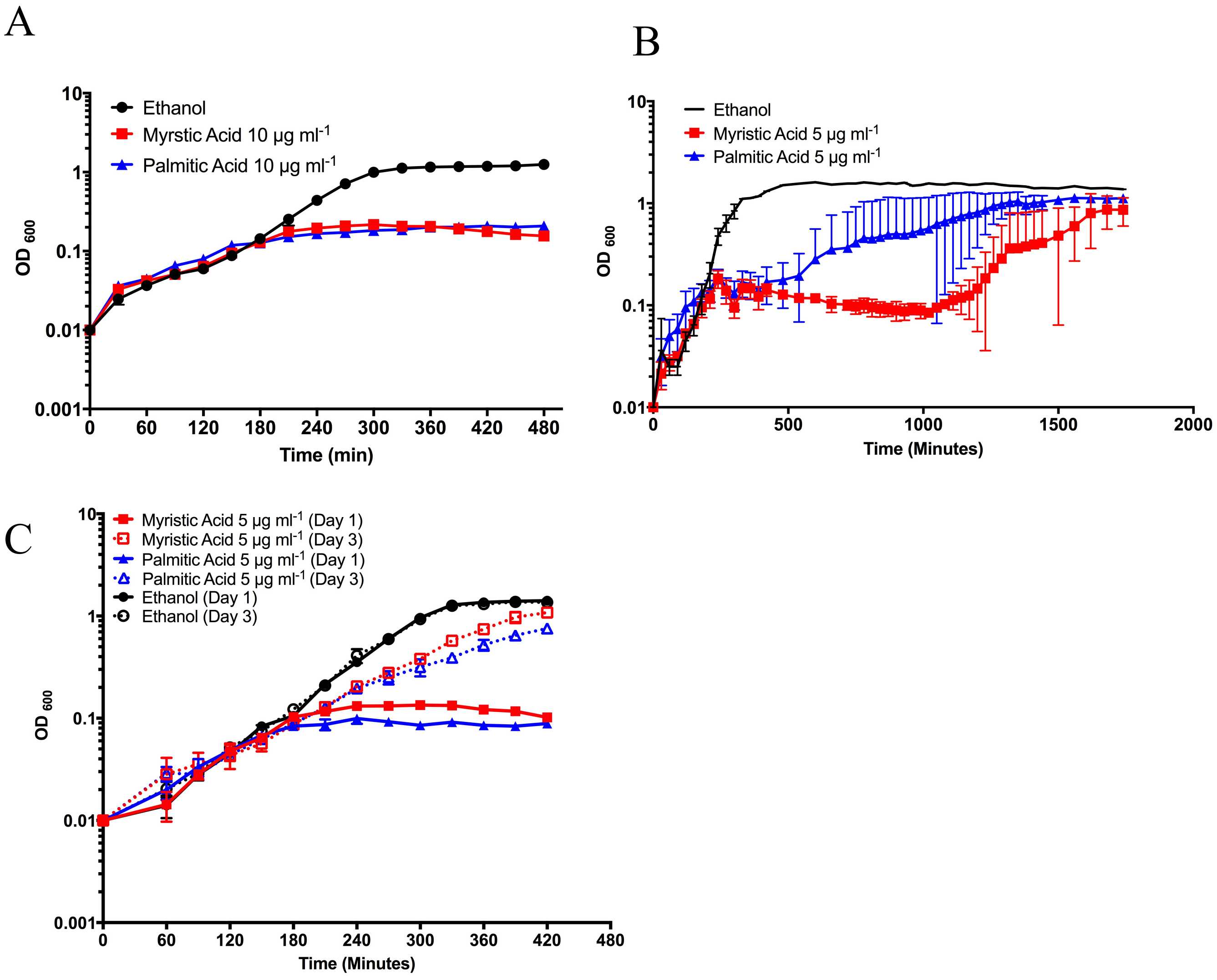

FIG S1 (A) OG1RF grown with either 10 µg ml^{-1} of myristic acid ($C_{14:0}$) or palmitic acid ($C_{16:0}$). (B) Outgrowth of OG1RF in BHI supplemented with 5 µg ml^{-1} of myristic acid ($C_{14:0}$) or palmitic acid ($C_{16:0}$). (C) OG1RF was diluted in medium containing the indicated supplements and growth was monitored over time (day 1). Following growth on day 1, cultures were diluted into medium lacking any fatty acid supplements and grown for 24 hours (day 2). On day 3, cultures were diluted into medium containing the indicated supplements and growth was monitored. Averages and standard deviations are shown for n= 3.

A

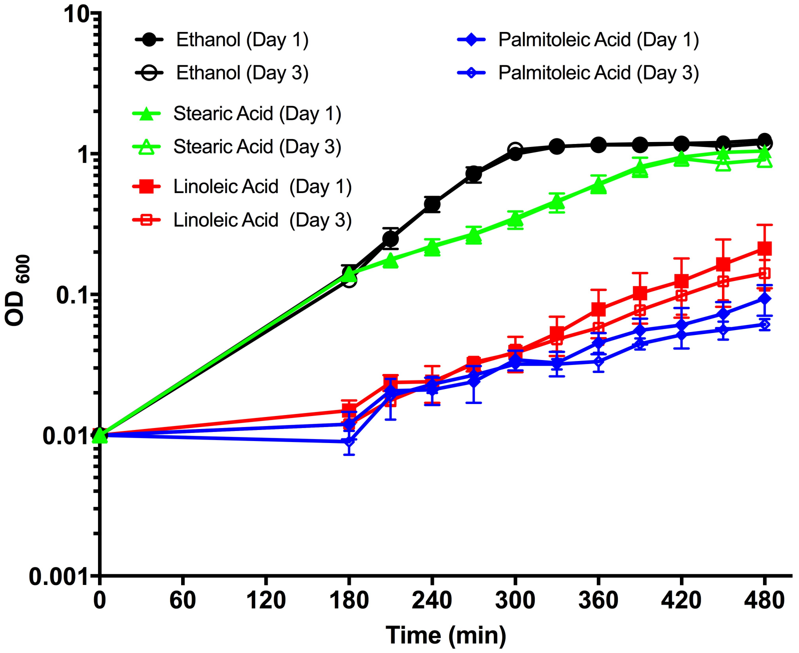

B

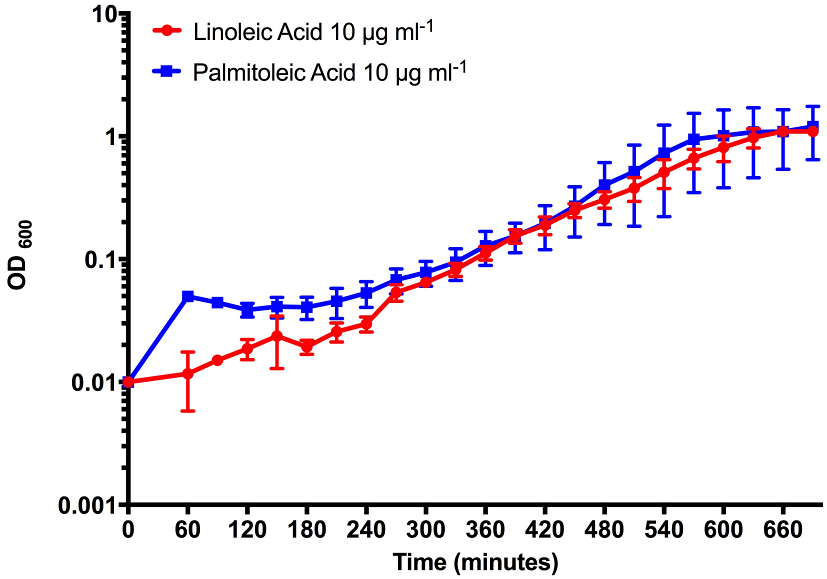

FIG S2 (A) OG1RF was diluted in medium containing either 20 μg ml^{-1} of stearic acid ($C_{18:0}$), the indicated supplements and growth was monitored over time (day 1). Following growth on day 1, cultures were diluted into medium lacking any fatty acid supplements and grown for 24 hours (day 2). On day 3, cultures were diluted into medium containing the indicated supplements and growth was monitored. Averages and standard deviations are shown for n= 3.

(B) Growth with 10 μg ml^{-1} linoleic acid or palmitoleic acid . Averages and standard deviations are shown for n= 3.

A B

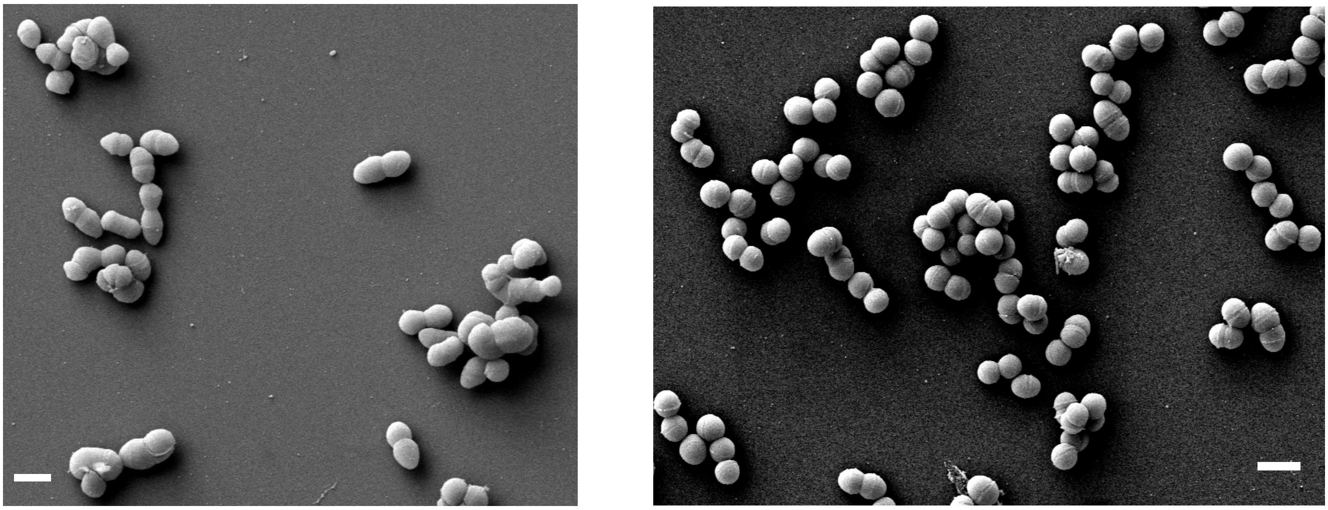

FIG S3 Scanning electron microscope images of *E. faecalis* during long term growth with fatty acid supplements. Fatty acids were added to a final concentration of 10 $\mu g\ ml^{-1}$ unless indicated. (A) Stearic acid: note the appearance of lancet-shaped cells, in addition to cells with distorted morphology. (B) Palmitoleic acid: note the uniform appearance of rounded cells throughout the population. White bar represents 1 μm. Shown are representative images from n= 3 experiments, minimum 10 fields observed per biological replicate.

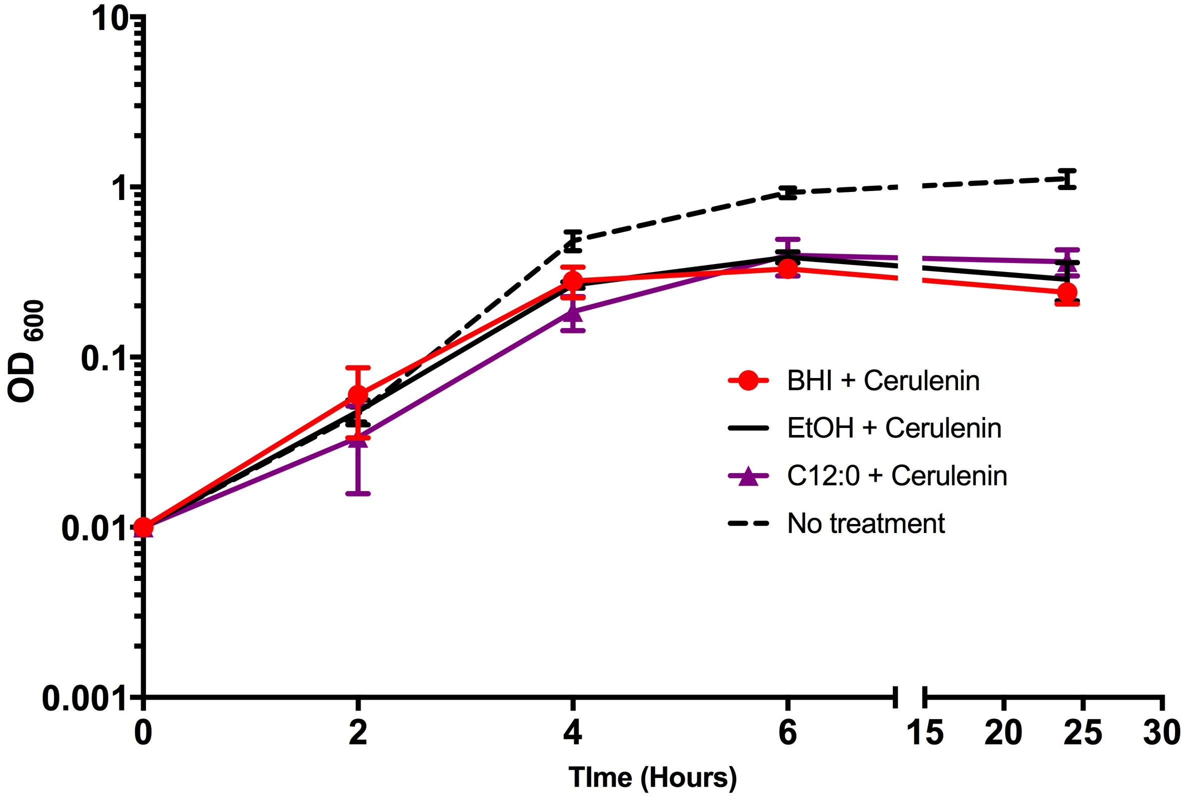

FIG S4 OG1RF cannot overcome cerulenin inhibition when provided with Supplementation with 10 μg ml^{-1} lauric acid. Overnight cultures of OG1RF were diluted to OD 600 nm 0.01 in BHI medium with 5 μg mL^{-1} cerulenin, and either 5 μg ml^{-1} lauric acid or equivalent volume of ethanol as indicated. As a control, cells were also diluted back into medium lacking the inhibitor or fatty acids and is indicated as "no treatment." Shown are the averages and standard deviations of n=3.

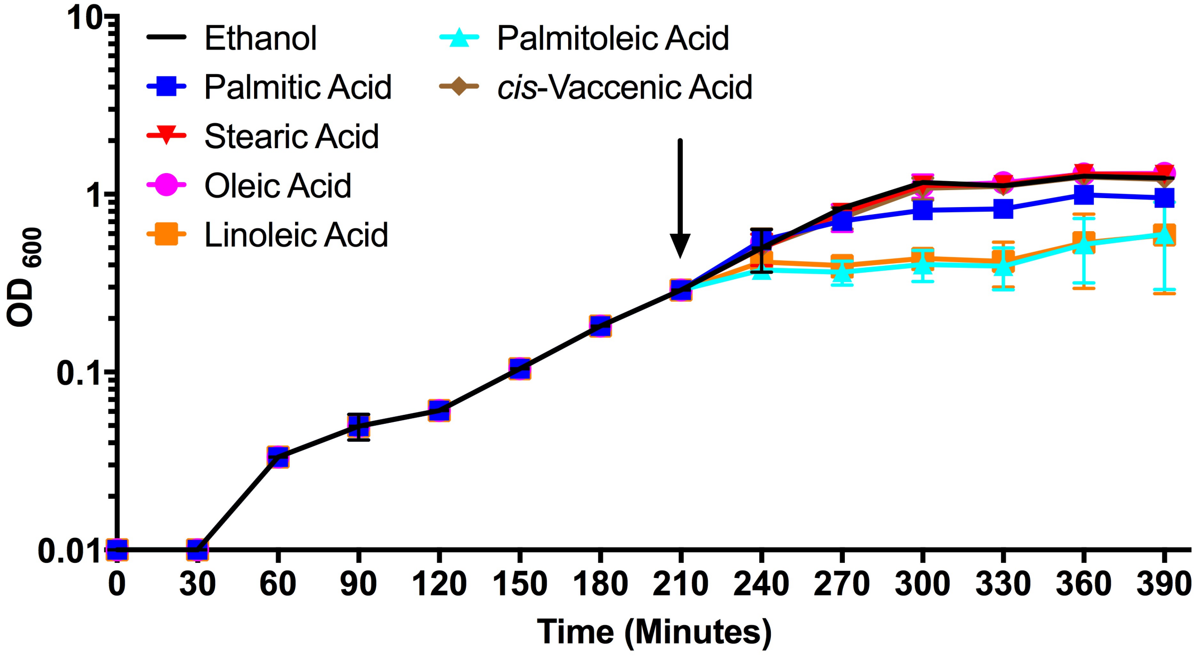

FIG S5 Growth of OG1RF following fatty acid addition during exponential phase. Cells were diluted into medium and grown until exponential phase at which point fatty acids (final concentration of 10 μg ml^{-1}) or solvent control were added (indicated by the arrow).
